# Genome-wide identification and expression analysis of PUB genes in cotton

**DOI:** 10.1186/s12864-020-6638-5

**Published:** 2020-03-06

**Authors:** Xuke Lu, Na Shu, Delong Wang, Junjuan Wang, Xiugui Chen, Binglei Zhang, Shuai Wang, Lixue Guo, Chao Chen, Wuwei Ye

**Affiliations:** 1 State Key Laboratory of Cotton Biology/ Institute of Cotton Research, Chinese Academy of Agricultural Sciences / Key Laboratory for Cotton Genetic Improvement, Anyang, 455000 Henan China; 2 Hanzhong Agricultural Science Institute, Hanzhong, 723000 Shanxi China

**Keywords:** U-box domain, PUB gene, Cotton, Evolution, Collinearity, Homologous gene pairs

## Abstract

**Background:**

The U-box gene encodes a ubiquitin ligase that contain U-box domain. The plant U-box gene (PUB) plays an important role in the response to stresses, but few reports about PUBs in cotton were available. Therefore research on PUBs is of great importance and a necessity when studying the mechanisms of stress- tolerance in cotton.

**Results:**

In this study, we identified 93, 96, 185 and 208 PUBs from four sequenced cotton species *G. raimondii* (D_5_), *G. arboreum* (A_2_), *G. hirsutum* (AD_1_) and *G. barbadense* (AD2), respectively. Prediction analysis of subcellular localization showed that the PUBs in cotton were widely localized in cells, but primarily in the nucleus. The PUBs in cotton were classified into six subfamilies (A-F) on the basis of phylogenetic analysis, which was testified by the analysis of conserved motifs and exon-intron structures. Chromosomal localization analysis showed that cotton PUBs were unevenly anchored on all chromosomes, varying from 1 to 14 per chromosome. Through multiple sequence alignment analysis, 3 tandem duplications and 28 segmental duplications in cotton genome D_5_, 2 tandem duplications and 25 segmental duplications in A_2_, and 143 homologous gene pairs in A_2_ and D_5_ were found; however no tandem duplications in A_2_ or D_5_ were found. Additionally, 105, 14 and 17 homologous gene pairs were found in the intra-subgenome of At and Dt, At sub-genome and Dt sub-genome of *G. hirsutum*, respectively. Functional analysis of *GhPUB85A* and *GhPUB45D* showed that these genes positively responded to abiotic stresses, but the expression patterns were different. In addition, although the expression levels of these two homologous genes were similar, their contributions were different when responding to stresses, specifically showing different responses to abiotic stresses and functional differences between the two subgenomes of *G. hirsutum*.

**Conclusions:**

This study reported the genome-wide identification, structure, evolution and expression analysis of PUBs in cotton, and the results showed that the PUBs were highly conserved throughout the evolutionary history of cotton. All PUB genes were involved in the response to abiotic stresses (including salt, drought, hot and cold) to varying degrees.

## Background

The ubiquitin-mediated ubiquitination pathway is the post-translational modification pathway of eukaryotic proteins. Studies have demonstrated that the pathway is involved in the cell cycles of higher plants [1], stress resistance [2], signal transduction [3], apoptosis [4], optical signal [5] and other physiological pathways. In the ubiquitin pathway, three steps are required for ubiquitin to act on the target protein. First, the ubiquitin-activating enzyme (E1) activates ubiquitin [1], and then the activated Ub molecules are delivered to the ubiquitin-binding enzyme (E2) [2]; finally, the Ub molecules are transferred to the target protein through ubiquitin ligase (E3) to work. In the pathway, E3 is critical for the identification of the specific substrate protein and can be found in the most species [3]. In *Arabidopsis*, there are more than 1400 genes encoding functional components of the ubiquitination pathway, of which approximately 90% genes were related to ubiquitin ligase E3 [3, 4]. Based on the composition of subunits and functional mechanism, the ubiquitin ligase E3 can be categorized into single-subunit type, such as HECT, RING/U-box [5] and multi-subunit type, such as SCF (skp1-cullin-F-box), APC (anaphase-promoting complex) [6], VBC (VHL-Elongin B-Elongin C) [7, 8], etc.

PUBs have been reported in many model crops, including *Arabidopsis*, rice, *Chlamydomonas reinhardtii*, Chinese cabbage, and soybean. Previous studies reported 64 PUBs were identified in *Arabidopsis* [9], 77 in rice [10], 33 in *Chlamydomonas reinhardtii* [11], 101 in Chinese cabbage [12] and 125 in soybean [13], indicating that PUB genes are widely distributed in plants. Many studies have shown that PUB proteins are involved in abiotic stress responses. Cho et al. obtained U-box E3 protein (CaPUB1) from water-stressed hot pepper plants and found *CaPUB1*-overexpressing plants displayed increased sensitivity water stress and mild salinity [14]. In *Arabidopsis*, proteins AtPUB22 and AtPUB23 were all negatively involved in the drought response by synergistic ubiquitination of RPN12a [15]. Liu et al. identified a U-box E3 protein AtPUB19 which was ep-regulated by drought, salt, cold and ABA. Down-regulation of *AtPUB19* led to hypersensitivity to ABA, enhancing ABA-induced stomatal closing, and drought tolerance, while overexpression of AtPUB19 resulted in the reverse phenotypes [16]. Previous studies also showed that the drought resistance *of OsPUB15*-overexpressing plants was significantly enhanced, and *OsPUB15* could be induced by hydrogen peroxide, drought and salt, indicating that *OsPUB15* positively regulated the drought response by attenuating intracellular oxidative stress [17].

Cotton is the most important fiber crop and the model crop for research into polyploidy, evolution, cell wall development, and cellulose synthesis [18]. Approximately 50 cotton species were distributed in arid and semi- arid regions of the tropic and subtropics, which were presumed to have originated from the same ancestor 50 to 100 million years ago [19]. The current cultivars are diploid *G. arboreum* and *G. herbaceum*, and tetraploid *G. hirsutum* and *G. barbadense*. The tetraploid cottons originated from the hybridization of an African ancestral species with A genome and an American ancestor species with D genome one to two million years ago [18]. Recently the sequencing work of diploid cottons *G. raimondii* (D5) [20, 21] and *G. arboreum* (A2) [22], and allotetraploid cottons *G. hirsutum*tm-1 (AD1) [23, 24] and *G. barbadense* acc.3–79 (AD2) [25] were completed, providing references for the study of gene function and evolution at the whole genome level. Based on the cotton genome sequences, the research about the genome-wide identification, structure, evolutionary relationship and expression analysis of PUBs would be well conducted, and this could provide some evaluable information for the functional analysis of PUBs in in cotton in the future.

## Results

### Genome-wide identification of PUB gene family members in cotton

The hidden Markov model (HMM) of the U-box domain (PF04564) was downloaded from the Pfam30.0 database, and used as a query to identify the candidate PUB members in four cotton genomic database using HMMER3.0. SMART. In addition, Pfam30.0 was also used for further identification to confirm every PUB members containing U-box domain. Finally, 93, 96, 185, and 208 PUBs were identified from the four sequenced cotton species *G. raimondii* (D_5_), *G. arboreum* (A_2_), *G. hirsutum* acc. TM-1 (AD_1_), and *G. barbadense* (AD2), respectively, and these PUBs were named *GrPUB1–93*, *GaPUB1–96*, *GhPUB1A-89A*/*1D-91D*/*181–185* and *GbPUB1A-98A*/*1D-98D*/*197–208* according to their location on the chromosome. The number of PUB genes in tetraploid cottons was twice as high as that in diploid cottons, showing that PUB genes were relatively conservative. The essential information about the gene name, chromosome locations, length of the open reading frame (ORF), type of protein domain, position of the U-box domain and subcellular localizations of these gene family members could be found in additional files (Additional file 2: Table S2, Additional file 3: Table S3, Additional file 4: Table S4 and Additional file 5: Table S5). The length of the PUB protein sequence in the cotton ranged from 49 to 1492 AA, and the U-box domain contained approximately 75 amino acids. However, the length of the U-box domain was almost identical except for a few PUBs; for example, proteins GaPUB39 and GhPUB40D had only 32 and 50 amino acids, respectively. Results of the subcellular localization analysis showed PUB proteins could be found throughout the cell, including nuclear, cytoplasmic, chloroplast, plasma membrane, mitochondrial, and extracellular locations. However, most PUB proteins were localized inside the nucleus. Twenty different domains were found among all the cotton PUBs (Table 1), and the primary mode was “U-box+ARM/HEAT”. Different domain modes may be associated with different functions of cotton PUBs.

**Table 1:** Domain organizations of PUB proteins in cotton

No.	Composition of domain (from N end to C end)	Number of proteins			
		*G.raimondii*	*G.arboreum*	*G. hirsutum*	*G. barbadense*
1	UFD2 specific motif + U-box	1	1	2	2
2	U-box + ARM	43	45	78	90
3	U-box only	33	35	68	83
4	STYKc + U-box	3	3	6	7
5	S_TKc + U-box	3	3	6	6
6	U-box + Prp19 + WD40	3	2	3	6
7	U-box + WD40	2	2	6	3
8	TPR + U-box	1	2	3	3
9	U-box + PPIase	1	1	2	2
10	U-box + KAP	1	0	1	0
11	UspA + S_TKc + U-box	2	2	2	2
12	UspA + STYKc + U-box	0	0	2	1
13	NRAMP + U-box + ARM	0	0	2	0
14	Ank + U-box + ARM	0	0	1	0
15	Ank + U-box + ARM + Ank	0	0	1	0
16	RPOL_N + RNA_pol + U-box + ARM	0	0	1	0
17	U-box + WD40 + S_TKc	0	0	1	0
18	EGF + PAN_AP + U-box + ARM	0	0	0	1
19	U-box + Arm + DNA_pol3	0	0	0	1
20	Pkinase + U-box	0	0	0	1

### Structure and evolution analysis of PUBs in cotton

A Gene structure diagram of the PUBs and an evolution tree were constructed (Additional file 6: Fig. S1, Additional file 7: Fig. S2, Additional file 8: Fig. S3 and Additional file 9: Fig. S4). Based on the evolutionary relationship, the PUB genes could be categorized into five subgroups (I-V). Among these subgroups, subgroup I was composed of the domains “U-box + ARM” and “U-box only”, and the remaining subgroups were composed of the other domains. The exon number of PUB genes in cotton was greatly divergent, ranging from 1 to 25. Among all the PUBs, approximately 1/3 of the PUBs contained only one exon. Generally, the evolutionary relationship is correlated with gene structure in some way, that is, exons with the more similarities in terms of the number and size of the exon, have a closer evolutionary relationship. In *G. hirsutum*, the length of *GhPUB1A* is 47 Kb, much larger than the other PUB genes, which may be correlated with the assembly and annotation of the cotton genome. Members in each subgroup of *G. barbadense* (AD2) was much different with those in *G. raimondii* (D_5_), *G. arboreum* (A_2_) and *G. hirsutum* (AD_1_), and this difference may be correlated with the different origins of these species. Therefore, the PUBs in *G. raimondii* (D_5_), *G. arboreum* (A_2_) and *G. hirsutum* (AD_1_) were used for the evolution relationship analysis, and the results also indicated five subgroups namedi-vwere found (Additional file 10: Fig. S5), and this was similar with the evolution of PUBs in one genome, indicating the PUB members were highly conservative. Furthermore, closer evolution relationships of GhPUB1A-89A with GaPUB1–96 and GhPUB1D-91D with GaPUB1–93 were found through the evolutionary analysis.

### Chromosomal localization analysis of PUB genes in three cotton genomes

**Fig. 1:**
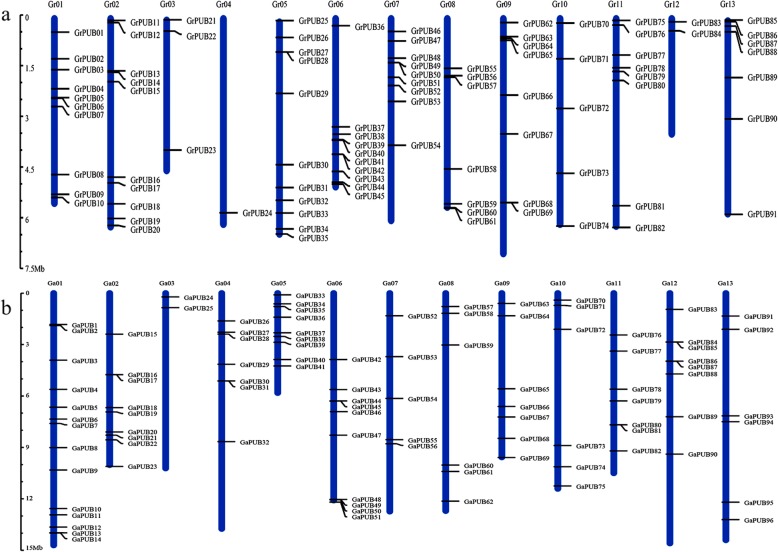
Locations of PUB genes on chromosomes in *G. raimondii* and *G. arboreum.* **a**, Locations of PUB genes on chromosomes in *G. raimondii*; **b**, Locations of PUB genes on chromosomes in *G. arboreum*. Ga01-Ga13 and Gr01-Gr13 represent the chromosome 1 to chromosome 13 in *G. arboretum* and *G. raimondii*, respectively

**Fig. 2:**
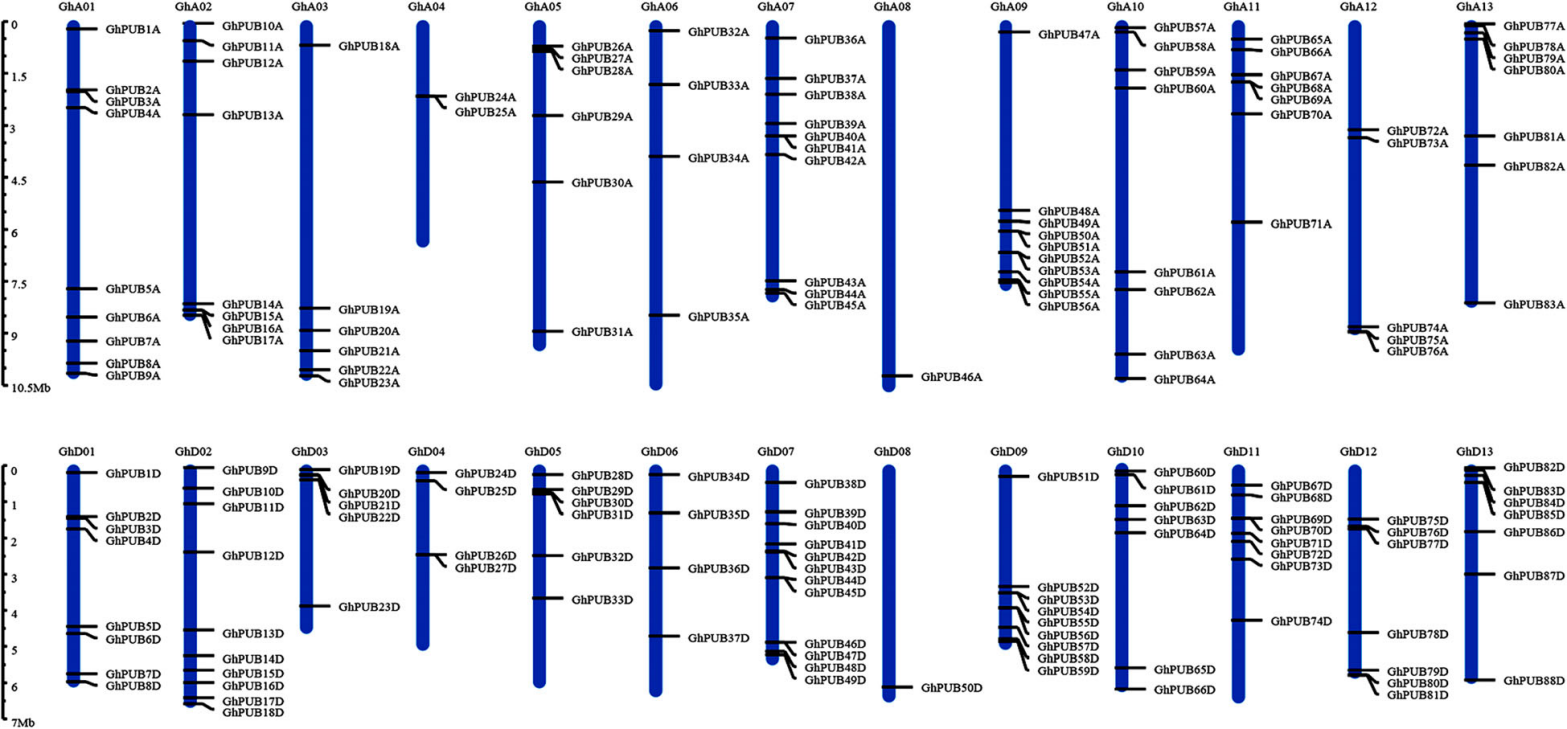
Locations of PUB genes on chromosomes in in *G. hirsutum.* Replace the chromosome 1 to chromosome 13 of A subgenome of *G. hirsutum* with GhA01-GhA13, Replace the chromosome 1 to chromosome 13 of D subgenome of *G. hirsutum* with GhD01-GhD13

The MapInspect software was used to analyze the localization of PUB genes on the chromosomes based on the position information. Among 93 genes in *G. raimondii*, 91 were localized unevenly on the chromosome and the others were found on scaffolds (Fig. 1a). These results indicated that only a few genes were present on chromosomes 3, 4, and 12, and the chromosome 5 contained the highest number of PUB genes (11 PUBs). In addition, PUB genes on chromosomes 4, 6, 7, 11 and 12 were preferentially enriched towards the end of the chromosome. All of 96 PUB genes identified in *G. arboreum* were localized on different chromosomes (Fig. 1b). The results showed uneven distribution of PUBs on each chromosome in *G. arboretum*, chromosome 1 containing the most PUB genes (up to 14) and chromosome 3 containing the least PUB genes (only 2). In addition, the length of chromosome 5 was approximately 6 Mb, however 9 PUB genes were found on it, presenting the highest distribution density. In *G. hirsutum*, 91.4% (169/185) of the PUB genes were anchored onto chromosomes, as shown in Fig. 2, among which 82 and 87 genes were found in the At- and Dt- subgenome, respectively. The number of PUB genes on chromosome D07 was the most and chromosome D08 was the least compared with other chromosomes in both At- and Dt- subgenomes of *G. hirsutum*, showing that PUBs on these two chromosomes were relatively conserved and significant for cotton growth. The situation for *G. barbadense* was different with that of *G. hirsutum* (Additional file 11: Fig. S6). These results indicated that the PUB genes were equally distributed in At- and Dt- subgenomes but unevenly localized on each chromosome, which may be correlated with the differentiation of these species.

### Gene duplication analysis

Fragment duplications in the genome region may result in the scattering of the gene family members. Compared with other eukaryotes, plants always have a higher rate of gene replication. Recent studies have shown that *G. raimondii* have had at least two complete genome-wide replicates [26]. The segregation of diploid cotton A genome and D genome occurred about 5–10 Myr years ago [18], and allotetraploid *G. hirsutum* was generated from the hybridization of diploid cottons and the number of chromosomes were doubled 1–2 Myr years ago. In the study, BLAST2.2.31+ (ftp://ftp.ncbi.nlm.nih.gov/blast/executables /blast+/LATEST/) was used for BLASTN and BLASTP (value 10) screening of homologous gene pairs from the cotton PUB genes identified. The uneven distribution of PUB genes on the chromosome may be correlated with the gene duplication or partial fragment replication events during the long evolutionary history of the cotton genome. Each time the replication event occurs, the entire genetic sequence of the cotton is doubled, and over time, these redundant genes are recombined or lost [23]. Previous studies have shown that gene duplication and post-segregation phenomena are two major driving forces of evolution [27]. Based on the multiple sequence alignment of the encoding sequences and the proteins in diploid cotton, 18 and 27 homologous gene-pairs were discovered with MCScanX [28] in *G. raimondii* (D5) (Additional file 12: Fig. S7A) and *G. arboreum* (A2) (Additional file 12: Fig. S7B), respectively. Among these homologous gene-pairs, 15 segmental duplications and 3 tandem duplications were found in *G. raimondii*, and 25 segmental duplications and 2 tandem duplications were found in *G. arboreum*. The relationship between these two diploid cottons and *G. hirsutum* was analyzed (Additional file 13: Fig. S8). Totally 197 homologous gene-pairs were found between *G. raimondii* and *G. hirsutum*, among which 58.89% (116/197) were located in the Dt-subgenome, and 191 homologous gene-pairs were found in both *G. arboreum* and *G. hirsutum*, among of which 55.50%(106/191) were located in the At-subgenome. All these results indicated that more than half of homologous genes in *G. hirsutum* were derived from the corresponding diploid cotton genomes. Furthermore, approximately 41.11–44.50% of these homologous genes were originated from other diploid genomes.

### Expression pattern analysis of PUB genes in cotton

Based on previous transcriptome data of the PUBs under different stresses (including salt, drought, hot and cold) in *G. hirsutum*, 117, 148 and 119 PUB genes were found with FPKM > 1 in roots, stems and leaves, respectively, displaying tissue specificity. Among all the PUB genes, approximately 21 non-expressed PUB genes were identified in three tissues, and they may be associated with other specific regulation functions. All the PUB genes were categorized into five subgroups (I, II, III, IV and V), and similar expression patterns were found among all PUB genes (Additional file 14: Fig. S9 and Additional file 15: Fig. S10). In subgroup I, 18 PUB genes with profound expression differences were discovered; in addition, other PUB genes in subgroup II- IV were found to have a consistent expression pattern under different stresses. However, 4 PUB genes (*GhPUB32A* - *GhPUB38D)* in subgroup V showed a small expression difference under different stresses.

**Fig. 3:**
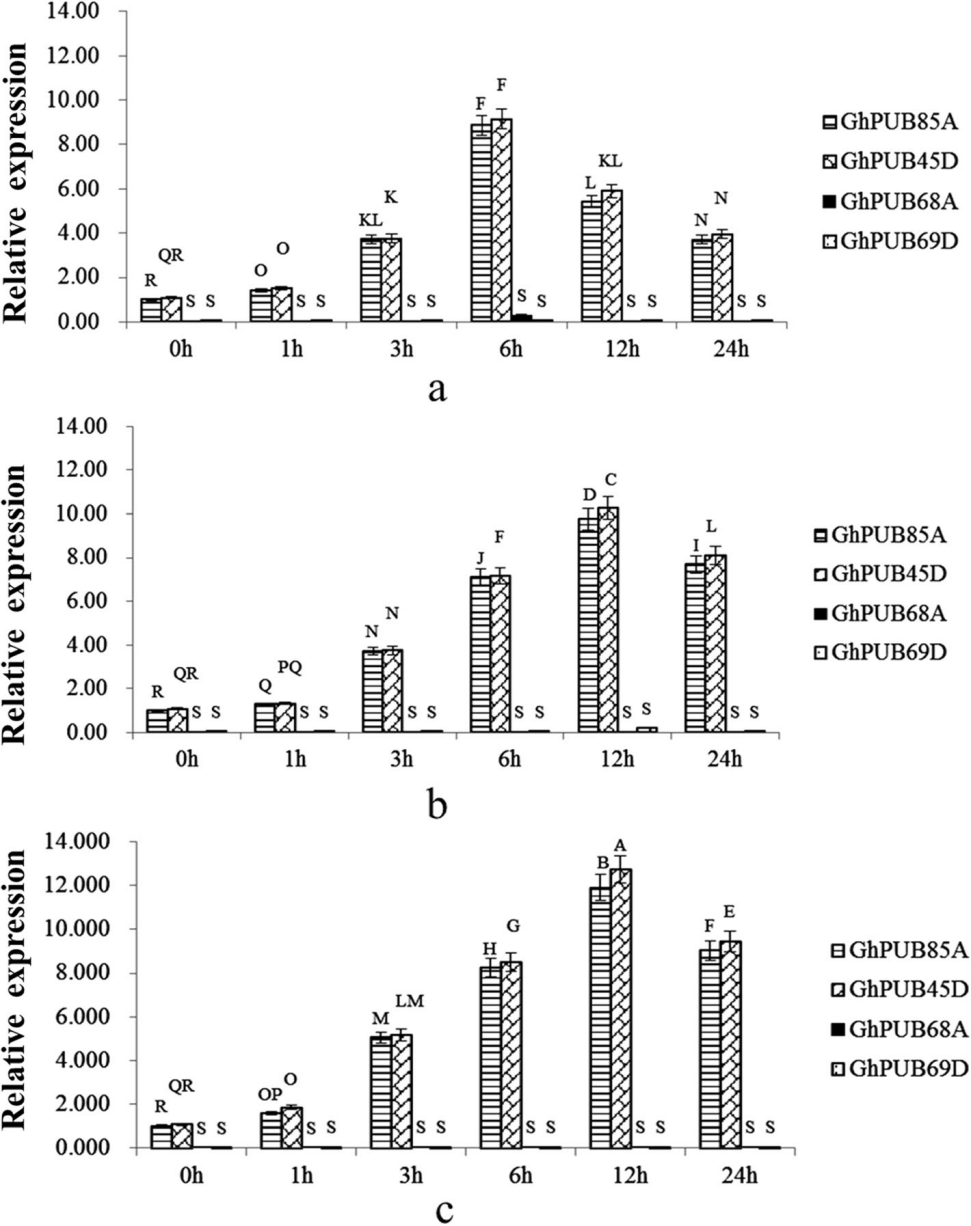
Expression patterns of *GhPUB68A*、*GhPUB85A*、*GhPUB45D* and *GhPUB69D* during the drought, salt or low temperature stress. **a**, **b** and **c** represent drought, salt and low-temperature treatment, respectively. Different letters from A to S indicate significance expression of different genes during different stresses (*p* < 0.01)

The evolution relationship in Additional 10: Fig. S5 showed *GhPUB68A*, *GhPUB85A*, *GhPUB45D* and *GhPUB69D* were belonged to subgroup III, indicating that their close relationship with each other. The transcriptome data showed that *GhPUB85A* and *GhPUB45D* were highly expressed whereas *GhPUB68A* and *GhPUB69D* were negligibly expressed. To investigate the functions of the homologous genes in cotton, qRT-PCR was used to investigate the expression difference in *G. hirsutum* TM-1. Drought, salt and cold treatments were applied and the results were present in Fig. 3. High expression of *GhPUB85A* and *GhPUB45D* under three stresses suggested that they were actively respond to the abiotic stresses, but *GhPUB68A* and *GhPUB69D* were not, which was in line with previously reported transcriptome data. Interestingly, we found that *GhPUB85A* and *GhPUB45D* were highly expressed at 6 h under drought stress, while the expressions at 12 h were the highest under salt and cold stress, indicating that *GhPUB85A* and *GhPUB45D* responded to drought stress faster than they did to salt and cold stresses. However, the expression values of *GhPUB85A* and *GhPUB45D* were significantly different under the same stress conditions, showing their different contributions in responding to abiotic stresses.

**Fig. 4:**
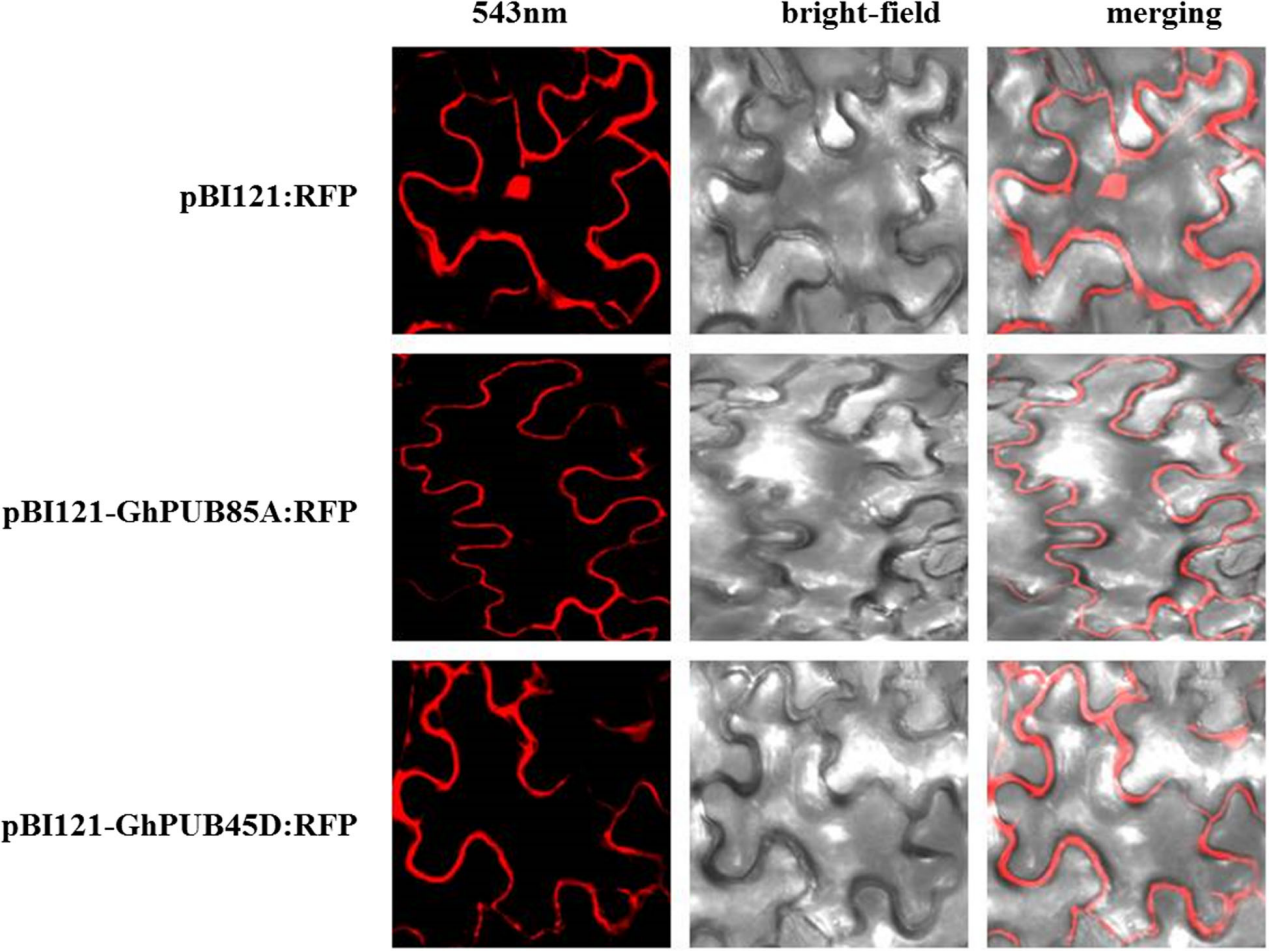
Subcellular localization of *GhPUB85A* and *GhPUB45D*

**Fig. 5:**
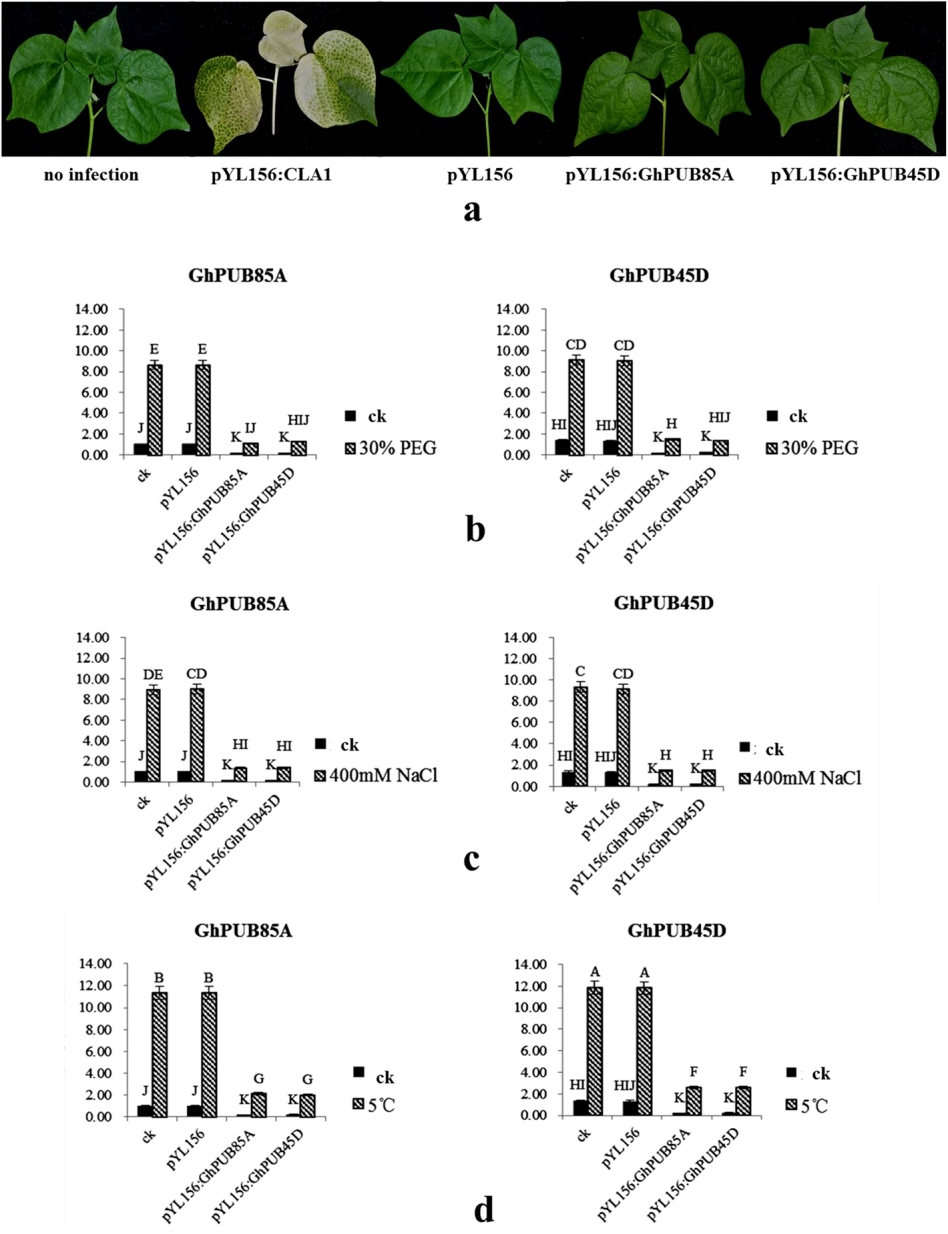
The phenotype of cotton leaves after virus infection and expression analysis of *GhPUB45D* and *GhPUB69D* under the drought, salt and low temperature stress. **a**, **b** and **c** represent drought (30% PEG), salt (400 mM NaCl) and low-temperature treatment (5 °C), respectively. Different letters from A to K indicate significance expression of different genes during different stresses (*p* < 0.01)

In addition, *GhPUB85A* and *GhPUB45D* were cloned using cDNA from *G. hirsutum* TM-1, and ligated to pEASY-Blunt Cloning Vector for sequencing to verify whether the vector was correctly ligated. The sequencing and enzyme digestion results showed that the recombined vectors were correctly constructed. Red fluorescence vectors pBI121-GhPUB85A:RFP and pBI121-GhPUB45D:RFP were constructed to research their subcellular localizations, and the results showed that these two genes were located at the cytomembrane, as shown in Fig. 4, which were consistent with our prediction in Additional file 2: Table S2. In addition, two VIGS vectors pYL156:GhPUB85A and pYL156:GhPUB45D were constructed using In-Fusion technology to study their functions under different stresses. Fifteen days after the VIGS infection, albino leaves of the positive control plants were observed, and all newly-emerged leaves were white in the later stage, while the others were normal with no albino leaves (Fig. 5a). We investigated the expression quantity in the control plants (CK), and pYL156-, pYL156:GhPUB85A- and pYL156:GhPUB45D- infected plants under different stresses. The expression levels of two genes decreased significantly after the VIGS infection under different treatments showed their positive functions in responding to multiple stresses and the success of VIGS infection (Fig. 5b-d), indicating the VIGS infection technology was an effective way to study the gene functions in cotton.

## Discussion

The PUB gene family has been identified and analyzed in a number of plants [10, 12–14]. In this study, bioinformatics analysis was performed on allotetraploid cotton genomes AD_1_ and AD2, and diploid cotton genomes A_2_ and D_5_, and finally a total of 582 PUB genes were identified, including 185 genes in AD_1_ genome, 208 in AD2 genome, 96 in A_2_ genome, and 93 in D_5_ genome, indicating that it was a relatively conserved family in terms of cotton genome evolution. Whole-genome replication analysis revealed that the ancestors of *G. arboreum*. and *G. raimondii* had undergone a cotton-specific genome-wide replication event that occurred about 1.6 million years ago after the differentiation from cocoa about 33 million years ago [29]. Recent studies have shown that *G. raimondii* has undergone at least two complete genome-wide replications [24, 30], resulting in an uneven distribution of the PUB genes on the chromosomes, and over the time of the cotton evolution, some genes are reassembled or lost. The results also showed that 19 of the 96 PUB genes in *G. arboreum* were generated through tandem repeats, which was one of the main reasons for the expansion of this gene family. Gene duplication event is a common phenomenon in plants, including multiple forms, such as tandem duplication, segmental duplication, and whole-genome duplication [31, 32]. Some of duplicated genes could be retained in its descendants, which could provide original genetic resource for adaptive evolution of plants [33]. In this research, gene duplication event was commonly found, totally 31 and 27 gene pairs were discovered in D5 and A2 genome, respectively. In *G. hirsutum*, more gene pairs were found than the sum of A2 and D5, which might be associated with the higher resistance and wider adaptability of *G. hirsutum*.

The classification of PUB protein differs from that of the other gene families - it depends not only on U-box homology but also on domains other than U-box domains [34]. The evolutionary relationship of PUB genes between different cotton species is close, and the genetic structure in cotton is highly conserved. During the process of cotton evolution, in addition to the U-box domain, some other domains retained the basic functions of the family and enriched the diversity of PUB genes. Gene structure analysis showed that exon number of PUB genes varied greatly from 1 to 25, which might be due to the directional evolution in the function and structure of PUB genes during the long evolutionary history. All PUB genes could be divided into five subgroups (I-V) in each species according to the evolutionary relationship, which was different with the classification of U-box containing genes in *C.reinhardtii* [11]. In the study, subgroup II and IV were found containing only 4 and 2 PUB genes, respectively. These findings suggested that the gene structures and evolutionary relationship of PUB family members were significantly different between different species. Previous studies have demonstrated that plant U-box containing genes are widely involved in stress responses, disease resistance and nutrient defect responses in plants [35–37]. In this study, 89% of PUB genes were differentially expressed in three tissues under salt, drought, cold and hot stresses, which also proved that PUB genes played important roles in abiotic responses. Twenty two PUB genes, including 18 genes from *GhPUB8A* to *GhPUB51D* in subgroup I and 4 genes from *GhPUB32A* to *GhPUB38D* in subgroup V, showed significant expression differences under different abiotic stresses, suggesting that they evolved toward specific functions in the long history. This perspective was consistent with previous documents [38, 39].

Many studies have shown that PUB genes play an important role in the process of stress responses in plants. For example, the overexpression of *AtCHIP* gene in *Arabidopsis* resulted in its sensitivity to high temperature and low temperature [40], while the *AtPUB18*, *AtPUB19*, *AtPUB22* and *AtPUB23* responded positively to drought [16, 30, 35]. In this study, two homologous PUB genes *GhPUB85A* and *GhPUB45D* were discovered with the same length of ORFs, type of protein, and subcellular localizations. The only difference was their locations on chromosomes, *GhPUB85A* on chromosome A07 in At-subgenome while *GhPUB45D* on chromosome D07 in Dt-subgenome, which was not reported before. In addition, these two PUB genes *GhPUB85A* and *GhPUB45D* were cloned and functionally evaluated. Significant expression difference revealed that the functions of these homologous genes were similar in response to abiotic stresses, but their contributions differed from each other. Both of *GhPUB85A* and *GhPUB45D* contained the protein domain TPR, which was the same with *AtCHIP*, so we speculated that the functions of these two genes might be similar with *AtCHIP* [40], at least in response to cold stress which had been verified in the study. The results in this study laid a foundation for the further study of PUB genes of in cotton in future.

## Conclusions

Genome-wide identification and expression analysis of PUB family members in cotton in this manuscript provided insights into response mechanism to abiotic stresses. Although the PUBs were highly conserved throughout the evolutionary history of cotton, significant differences were found between each other in gene structure. In addition, two homologous genes *GhPUB85A* and *GhPUB45D* were cloned and functionally identified. Expression pattern analysis showed they both responded to abiotic stresses positively, but their contributions were different. Therefore all these results were of great significance for the future research of molecular mechanism in responding to abiotic stresses.

## Methods

### Planting of cotton and *Nicotiana benthamiana* seedlings

Upland cotton variety ZhongS9612, preserved by the Cotton Adversity Research Laboratory at the Chinese Academy of Agricultural Sciences (CAAS) for many years, was selected and used in the research. Before planting into sand, the seeds were surface-sterilized with 0.1% HgCl_2_ and placed in a sterile dish with moist filter paper to accelerate germination. Uniform seedlings were chosen and transplanted into sand pots (10 plants in each pot) in a greenhouse (14 h/day at 30 °C and 10 h/night at 24 °C) at the Institute of Cotton Research of CAAS. The cotton seedlings were treated with 200 ml 30% PEG 6000 and 200 ml 400 mM NaCl solution, which could achieve completely consistent stress environments for each seedling in one pot. For the cold treatment, cotton seedlings were transferred into a 5 °C refrigerator with clear glass, but the light condition was not changed. For the planting of *Nicotiana benthamiana*, we first placed the seeds on MS medium at 22 °C in a growth chamber with a 16-h light cycle for the germination. After the emergence, the tobacco seedlings were moved to nutrition-enriched soil for growth under the same condition for about 45 days. After the treatment, plant leaves were harvested and frozen with liquid nitrogen for use. For the agrobacterium-mediated transformation, we referred the method used by Lu et al. [41].

### Whole genome identification of PUBs in cotton

Cotton genome data (*G. raimondii* (D5) [20, 21], G. arboretum (A2) [22], and *G. hirsutum* acc. TM-1 (AD1) [23, 24] were obtained from CottonGene (https://www.cottongen.org/). The hidden Markov Model (HMM) profile of the U-box domain (PF04564) was obtained from Pfam30.0 (http://pfam.xfam.org/) [42], and was used as a query to identify the candidate PUBs from the cotton genome protein database using HMMER3.0 [43]. We used BLAST2.2.31+ (ftp://ftp.ncbi.nlm.nih.gov/blast/executables/blast+/LATEST/) to obtain the coding domain sequences (CDSs) sequences, protein sequences and the corresponding full-length sequence in the genome. The protein sequences were further analyzed in the SMART (http://smart.embl-heidelberg.de/) and Pfam 30.0 [42] databases to ensure that each candidate protein contained a U-box domain. A subcellular localization prediction was carried out in CELLO v.2.5 [44].

### Analysis of gene structure, phylogenetic relationship and conserved domain

All CDS sequences identified and the genome sequence of the PUBs were used to analyze the gene structure with software GSDS2.0 [26]. The full-length sequences of PUB proteins were used to construct a phylogenetic tree. Multi-sequence alignment of the PUBs was carried out by ClustalX1.83, and Neighbor-Joining (NJ) method [45] was used to construct a phylogenetic tree in MEGA6.0 [27]. The online software SMART, PROSITE [29] was used to analyze the conserved domains of each protein.

### Physical location of PUBs on the chromosome

GFF (general feature format) information of the cotton PUBs were obtained from the genome annotation files. The distribution of cotton PUBs on the chromosome was generated with MapInspect (http://mapinspect.software. informer. com/).

### Gene duplication and micro-synteny analysis in *G. arboreum*, *G. raimondii* and *G. hirsutum* L

Homologous gene pairs were identified according to multiple sequence alignment results and the standard was described in previous studies [46, 47]. The collinearity of homologous genes was visualized with program Circos-0.69 [48] based on the homology between each species and their positions on the genome.

### Gene cloning and the construction of vectors

The first strand was synthesized according to the instructions of the TransScript One-step gDNA Removal and cDNA Synthesis Supermix kit. Two homologous genes *GhPUB85A* and *GhPUB45D* were cloned using In-fusion technology. Primers were designed using the online software and sequence information was present in Additional file 1: Table S1**.** The PCR amplification products were verified using 1.5% agarose gel electrophoresis. PCR amplification products were purified using the MiniBEST Agarose Gel DNA Extraction Kit from Takara Corporation. Finally the concentration of targets was measured, and stored at − 20 °C for use.

Purified targets were linked to the pEASY-Blunt Cloning Vector, and then transformed into *E. coli* according to the instructions of pEASY-Blunt Cloning Kit. The transformation was conducted with heat-shock method, which was a classically effective approach. First a mixture of vectors and products was prepared with a mole ratio of 1:7 before the reaction in 200 μl centrifuge tube at 25 °C for 5 min. Second blend the linked products and *E. coli* DH5α cells when *E. coli* DH5α cells began to dissolve, then the reaction was performed on the ice for 30 min. Thirdly transfer the centrifuge tubes to 42 °C water for 90 s to end the reaction, at last placed the centrifuge tubes on the ice for use. Positive clones were selected and inoculated into LB liquid medium containing Kana (50 mg•L^− 1^) for about 6 h under the conditions of 200 rpm and 37 °C. Then the positive clones were verified using PCR amplification with primers. Finally cloning vectors GhPUB85A-t and GhPUB45D-t were obtained. PCR reaction system used in the research was below: 5 × PrimerSTAR GXL Buffer, 10.0 μl; dNTP Mixture (2.5 mM each), 4.0 μl; F-primer (10 μM), 1.5 μl; R-primer (10 μM), 1.5 μl; cDNA, 100 ng; 5 × PrimerSTAR GXL DNA Polymerase, 100 ng, 5 × PrimerSTAR GXL DNA Polymerase, 2.0 μl; add ddH_2_O to 50.0 μl. PCR procedure of gene amplification used in the research was: 98 °C, 10s; 55 °C, 15 s; 68 °C, 9 s; 4 °C, forever, 35 cycles. Real-time PCR method was used to measure the relative expression of two genes. Primers of two genes were listed in Additional file 1: Table S1. *GhHistone3* gene (AF02471) was used as the reference gene.

### VIGS analysis of GhPUB85A and GhPUB45D

Based on the vector sequence, target gene sequence and enzyme cutting sites, In-fusion primers were designed at the website (http://bioinfo.clontech.com/infusion). Primer sequences of InGhPUB85A-V, InGhPUB45D-V, GhPUBs-RFP and InGhPUBs were listed in Additional file 1: Table S1. Finally the silencing vectors pYL156:GhPUB85A and pYL156:GhPUB45D, and RFP vectors pBI121-GhPUB85A:RFP and pBI121-GhPUB45D:RFP, and the Plant overexpression vectors pBI121:GhPUB85A and pBI121:GhPUB45D were all successfully constructed. Vectors were transformed into cotton and tobacco with agrobacterium mediated genetic transformation method.

## Supplementary information

**Additional file 1 Table S1.** Primers used in the manuscript.

**Additional file 2 Table S2.** Essential information of PUB gene members in *Gossypium raimondii.*

**Additional file 3 Table S3.** Essential information of PUB gene members in *Gossypium arboreum.*

**Additional file 4 Table S4.** Essential information of PUB gene members in *Gossypium hirsutum* L.

**Additional file 5 Table S5.** Essential information of PUB gene members in *Gossypium barbadense.*

**Additional file 6 Fig. S1.** The phylogenetic relationship and gene structure analysis of GrPUBs in *G. raimondii.*

**Additional file 7 Fig. S2.** The phylogenetic relationship and gene structure analysis of GaPUBs in *G. arboreum.*

**Additional file 8 Fig. S3.** The phylogenetic relationship and gene structure analysis of GhPUBs in *G. hirsutum.*

**Additional file 9 Fig. S4.** The phylogenetic relationship and gene structure analysis of GbPUBs in *G. barbadense.*

**Additional file 10 Fig. S5.** The phylogenetic relationship analysis of PUBs in *Gossypium.*

**Additional file 11 Fig. S6.** Distrbution of GbPUBs on chromosomes in *G. barbadense.*

**Additional file 12 Fig. S7.** The homologous relationships of PUBs in *G. raimondii* and *G. arboreum.*

**Additional file 13 Fig. S8.** The intra- and inter-genomic synteny blocks of PUBs.

**Additional file 14 Fig. S9.** Predicted expression pattern of GhPUBs in upland cotton under salt and drought stress.

**Additional file 15 Fig. S10.** Predicted expression pattern of GhPUBs in upland cotton under cold and heat stress.

## Data Availability

All data generated and results analyzed during this study are included in this article and its supplementary information. Transcriptome data used for the PUB gene expression analysis in Additional file 14 Fig. S9 and Additional file 15 Fig. S10 could be downloaded with the accession number PRJNA248163.

## Abbreviations

APC: Anaphase-promoting complex
NJ: Neighbor-Joining
PUB: U-box gene
SCF: Skp1-cullin-F-box
VBC: VHL-Elongin B-Elongin C
