# Supplementary figures and images for: Genome-wide identification and expression analysis of PUB genes in cotton
Source: BMC Genomics. 2020 Mar 6;21:213. doi: 10.1186/s12864-020-6638-5 (PMC7060542; doi:10.1186/s12864-020-6638-5)

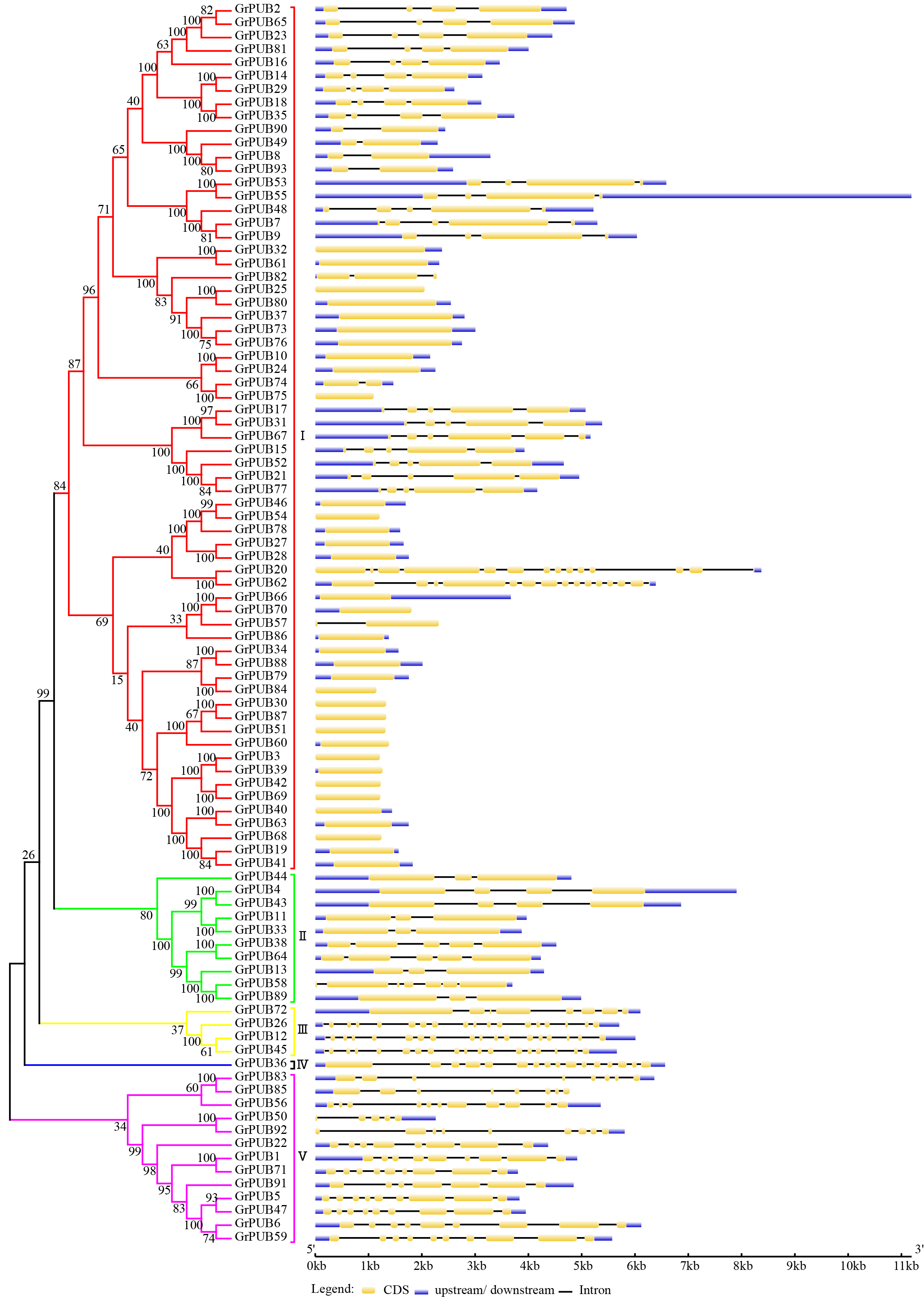

Supplement: Supplementary file 6 — Additional file 6 Fig. S1. The phylogenetic relationship and gene structure analysis of GrPUBs in G. raimondii. [file 12864_2020_6638_MOESM6_ESM.jpg]

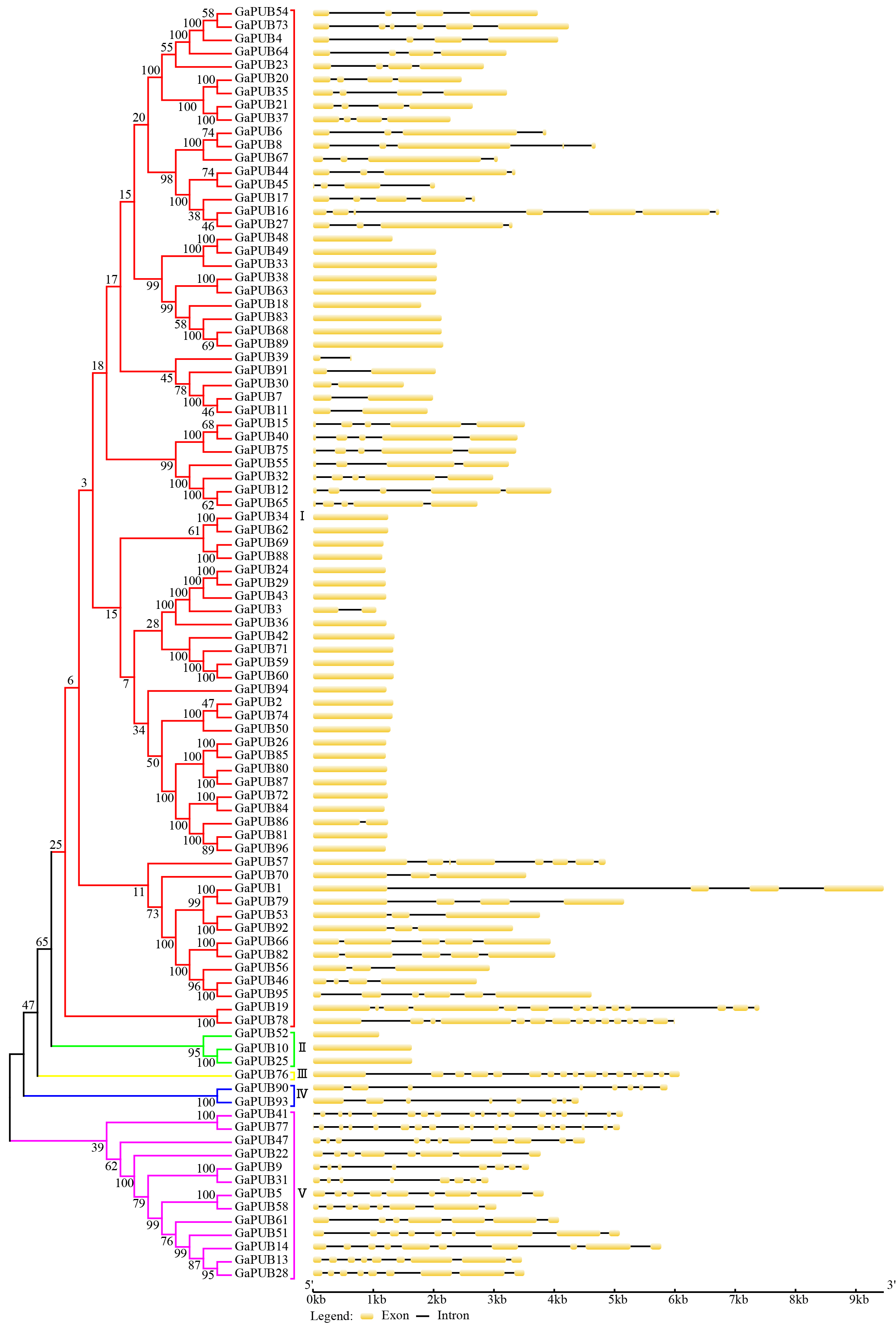

Supplement: Supplementary file 7 — Additional file 7 Fig. S2. The phylogenetic relationship and gene structure analysis of GaPUBs in G. arboreum. [file 12864_2020_6638_MOESM7_ESM.jpg]

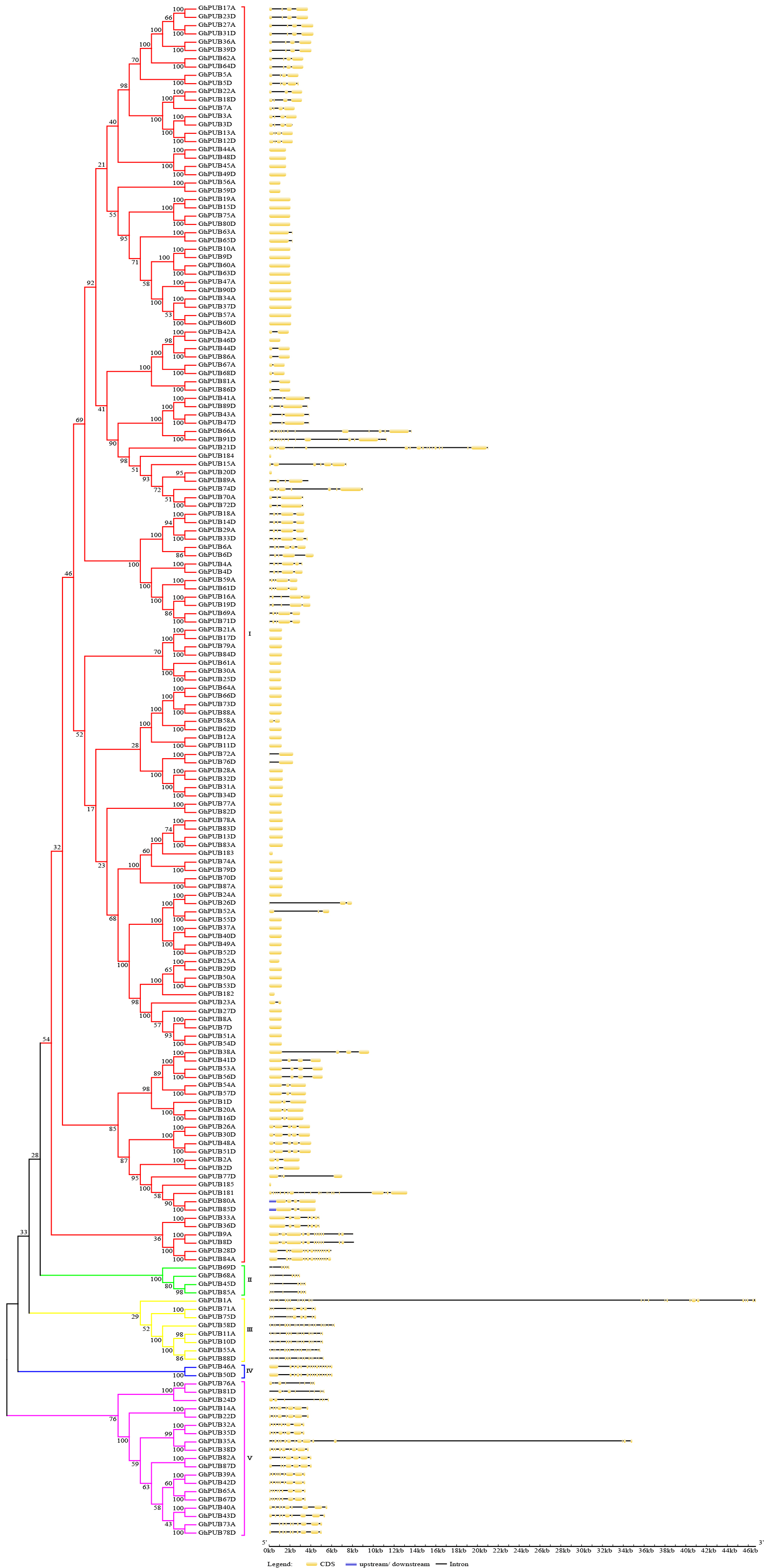

Supplement: Supplementary file 8 — Additional file 8 Fig. S3. The phylogenetic relationship and gene structure analysis of GhPUBs in G. hirsutum. [file 12864_2020_6638_MOESM8_ESM.jpg]

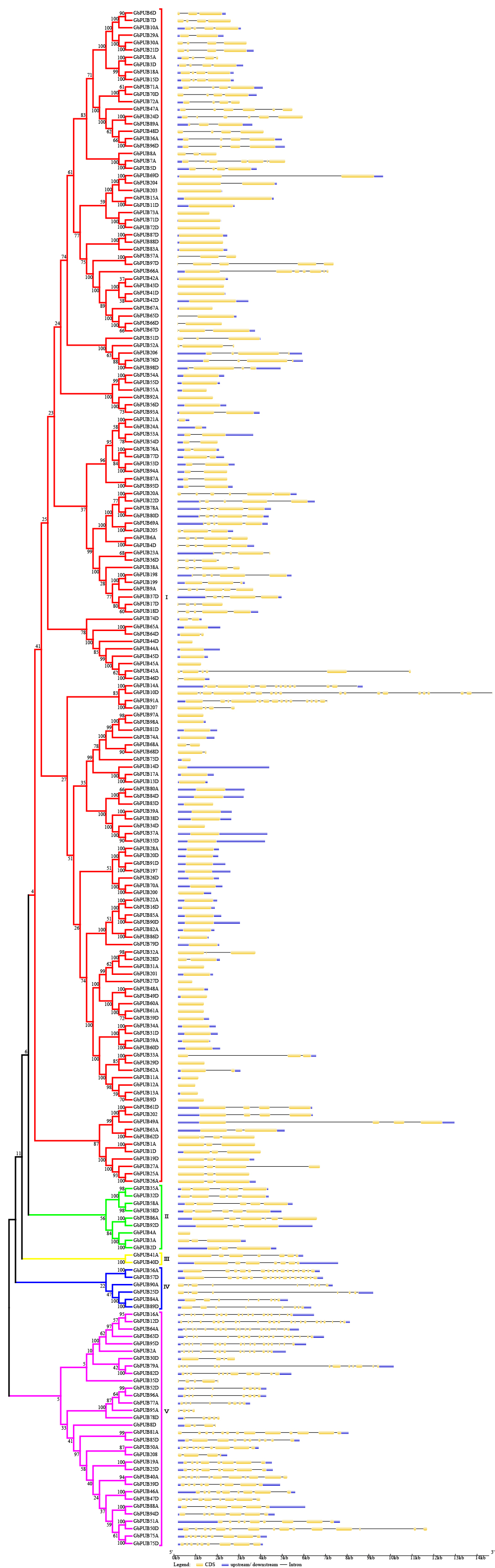

Supplement: Supplementary file 9 — Additional file 9 Fig. S4. The phylogenetic relationship and gene structure analysis of GbPUBs in G. barbadense. [file 12864_2020_6638_MOESM9_ESM.jpg]

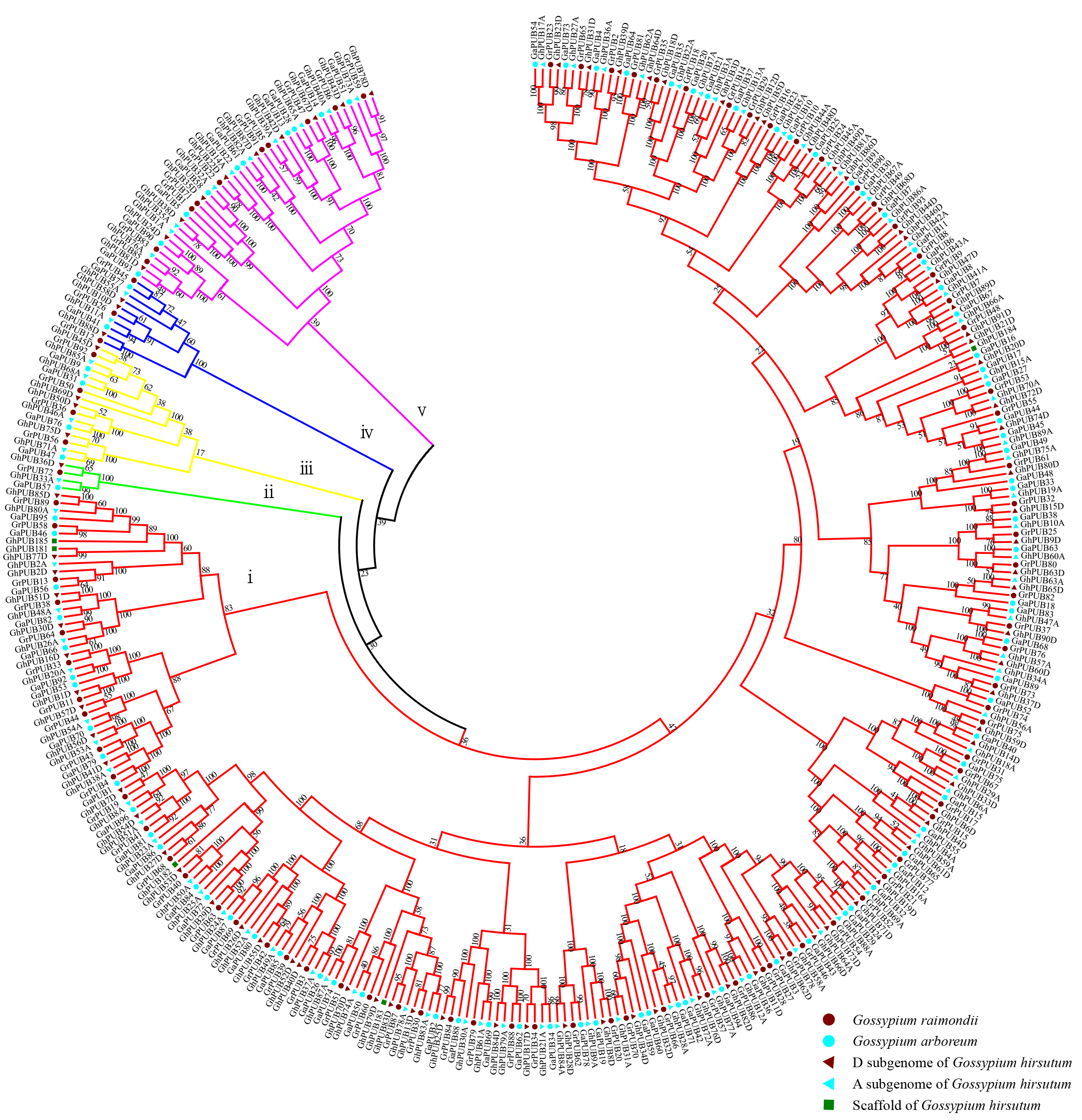

Supplement: Supplementary file 10 — Additional file 10 Fig. S5. The phylogenetic relationship analysis of PUBs in Gossypium. [file 12864_2020_6638_MOESM10_ESM.jpg]

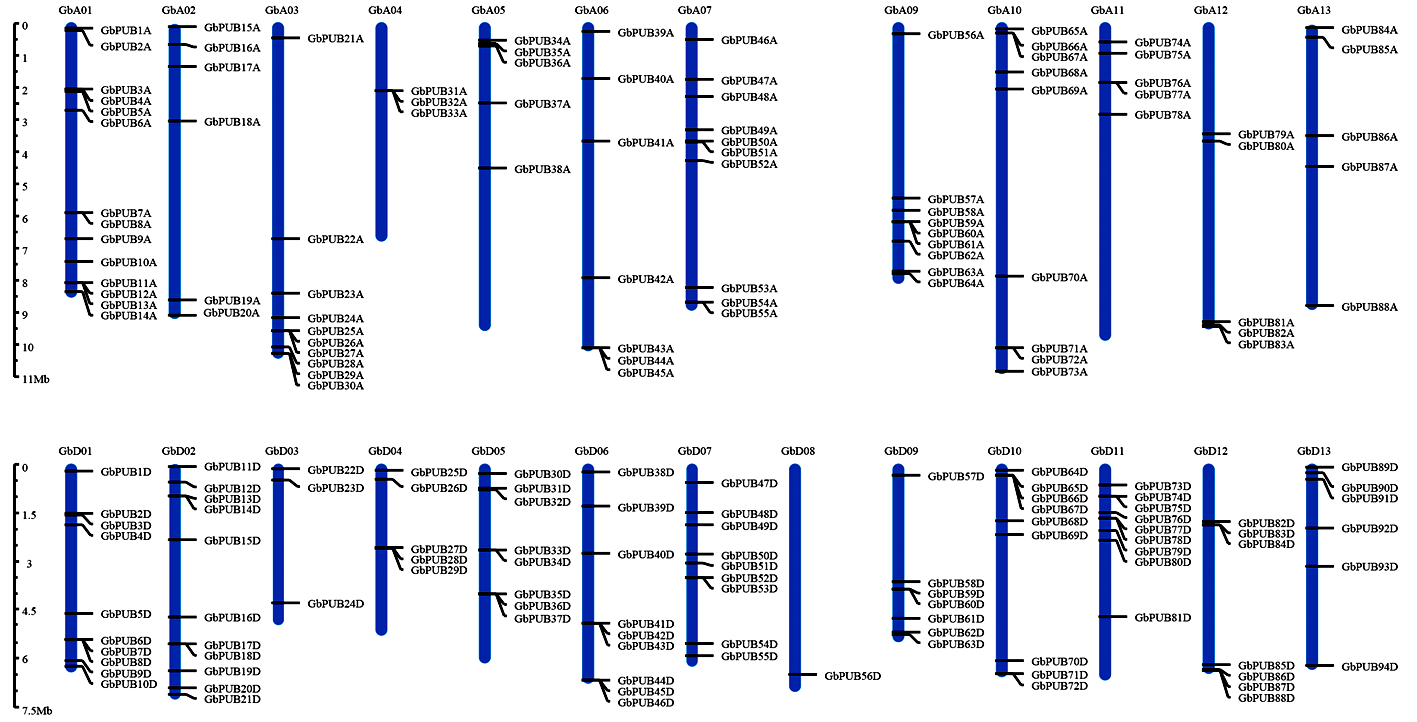

Supplement: Supplementary file 11 — Additional file 11 Fig. S6. Distrbution of GbPUBs on chromosomes in G. barbadense. [file 12864_2020_6638_MOESM11_ESM.tif]

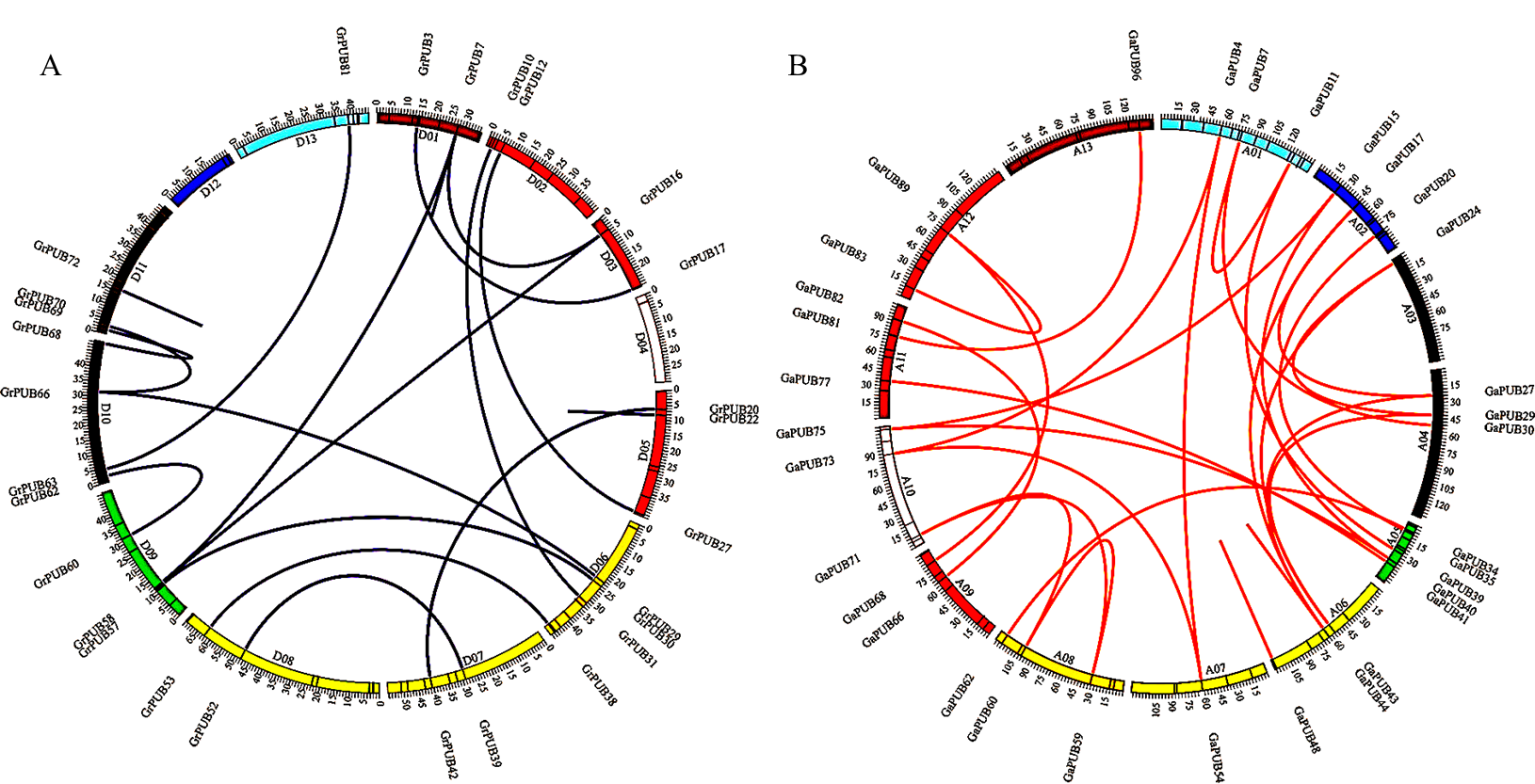

Supplement: Supplementary file 12 — Additional file 12 Fig. S7. The homologous relationships of PUBs in G. raimondii and G. arboreum. [file 12864_2020_6638_MOESM12_ESM.tif]

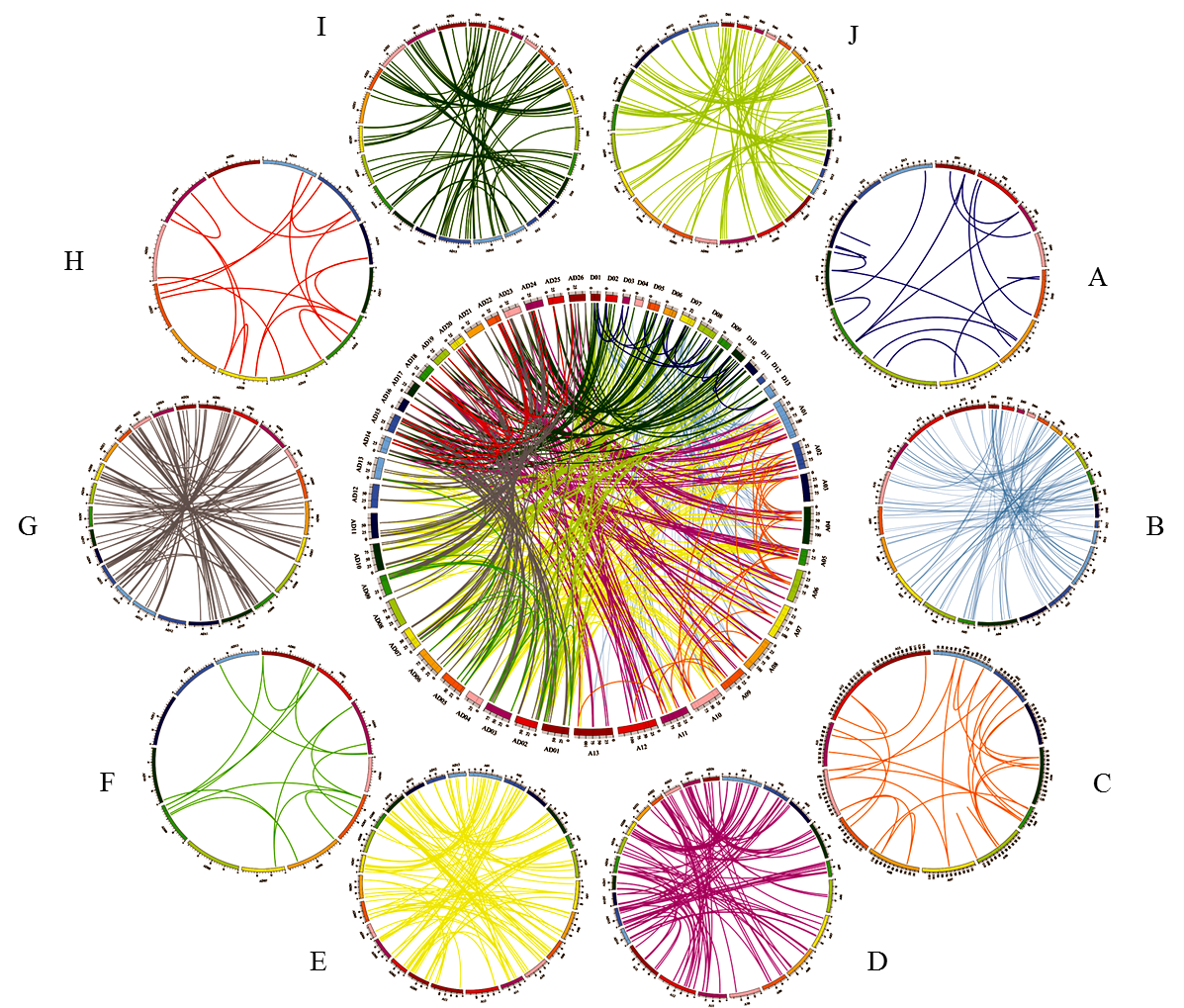

Supplement: Supplementary file 13 — Additional file 13 Fig. S8. The intra- and inter-genomic synteny blocks of PUBs. [file 12864_2020_6638_MOESM13_ESM.tif]

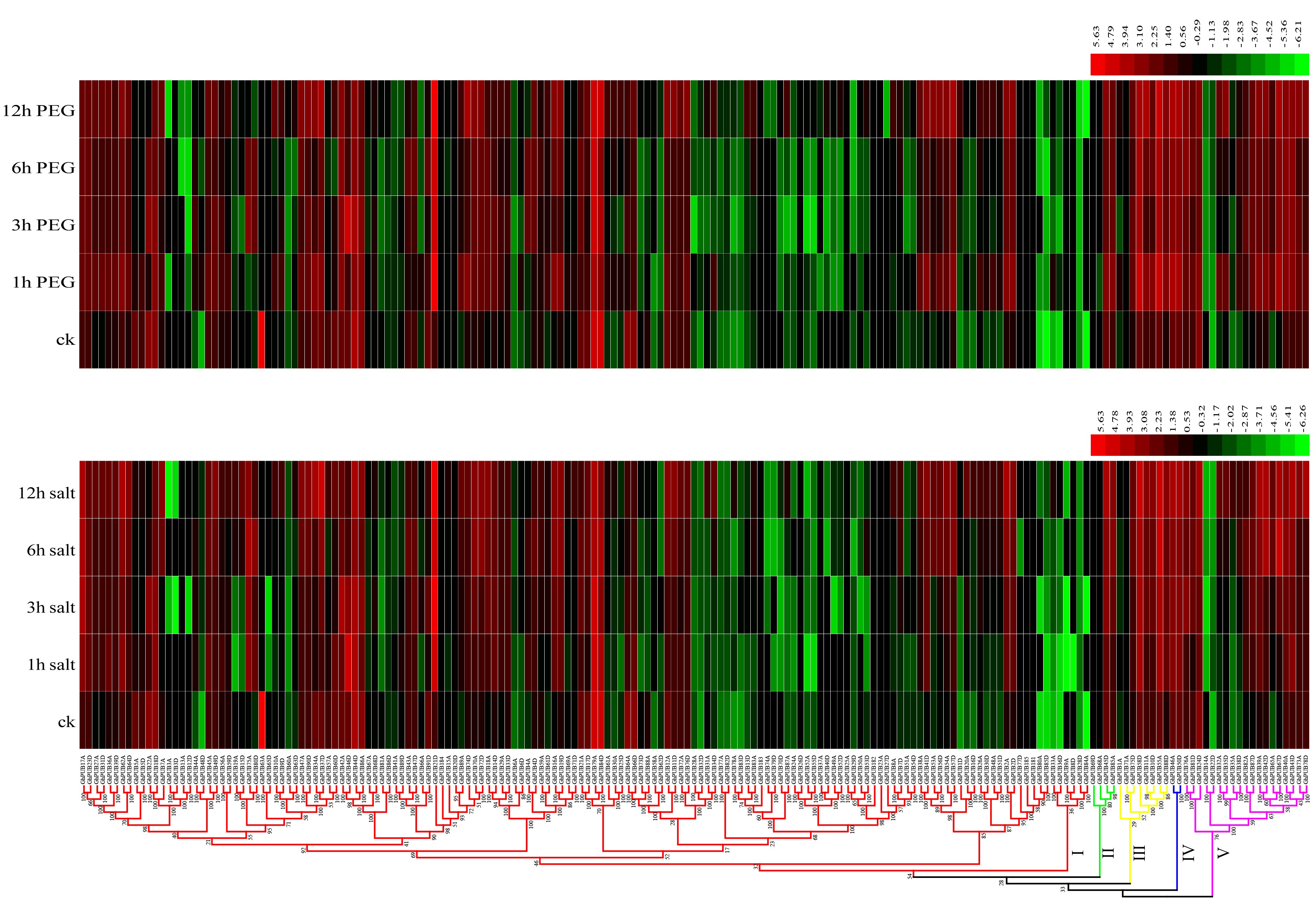

Supplement: Supplementary file 14 — Additional file 14 Fig. S9. Predicted expression pattern of GhPUBs in upland cotton under salt and drought stress. [file 12864_2020_6638_MOESM14_ESM.jpg]

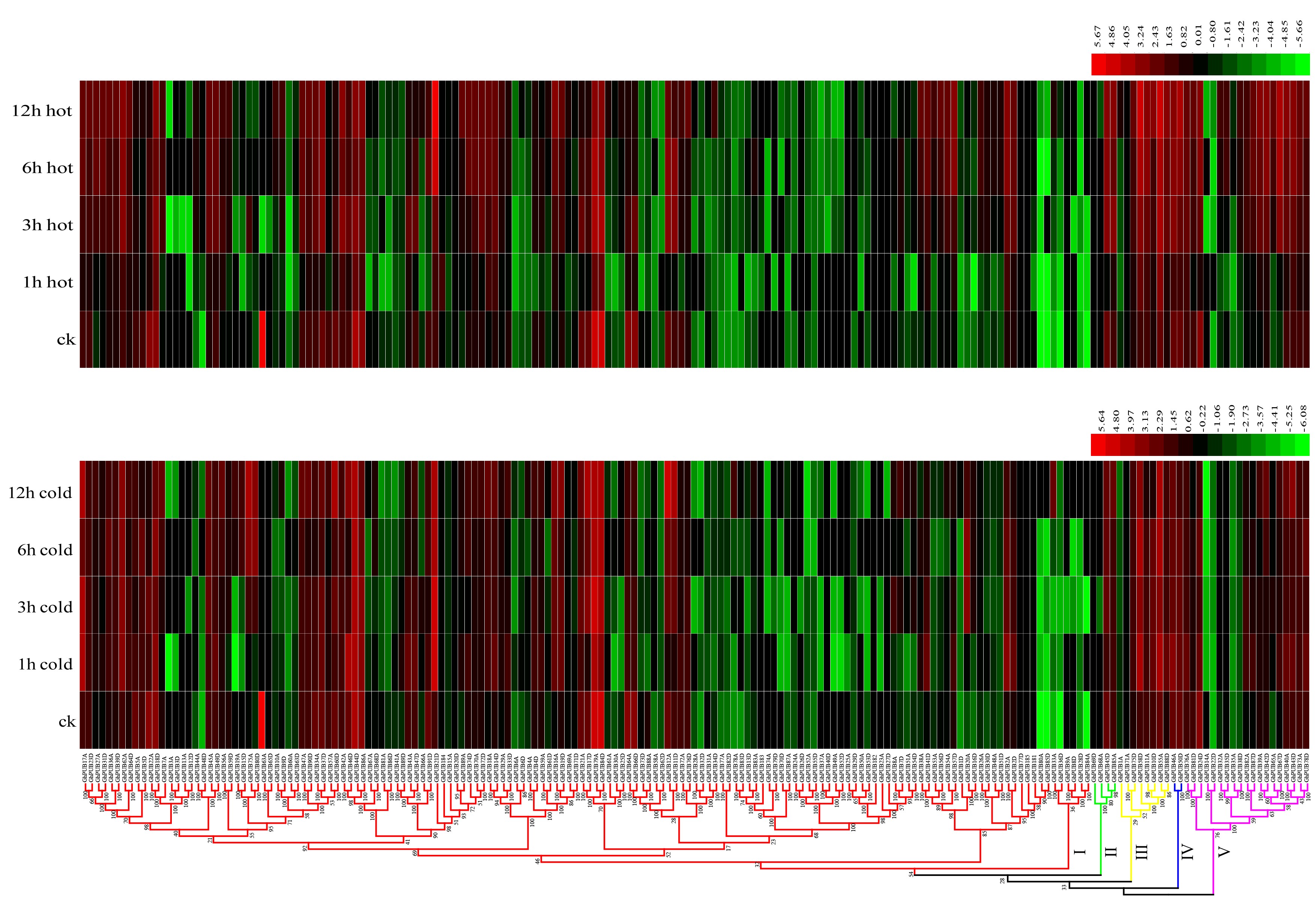

Supplement: Supplementary file 15 — Additional file 15 Fig. S10. Predicted expression pattern of GhPUBs in upland cotton under cold and heat stress. [file 12864_2020_6638_MOESM15_ESM.jpg]
